# Continuous Monitoring with AI-Enhanced BioMEMS Sensors: A Focus on Sustainable Energy Harvesting and Predictive Analytics

**DOI:** 10.3390/mi16080902

**Published:** 2025-07-31

**Authors:** Mingchen Cai, Hao Sun, Tianyue Yang, Hongxin Hu, Xubing Li, Yuan Jia

**Affiliations:** 1 School of Mechanical Engineering and Automation, Fuzhou University, Fuzhou 350108, China; 230227148@fzu.edu.cn (M.C.); huhongxin2025@163.com (H.H.); 2 School of Mechatronics Engineering, Harbin Institute of Technology, Harbin 150001, China; 3 State Key Laboratory of Robotics and System, Harbin Institute of Technology, Harbin 150001, China; 4 Faculty of Science and Technology, University of Macau, Macao SAR 999078, China; mc35301@um.edu.mo; 5 College of Intelligent Manufacturing, Honghe Vocational and Technical College, Honghe Hani and Yi Autonomous Prefecture 661100, China; baihuziyou@163.com; 6 Sino-German College of Intelligent Manufacturing, Shenzhen Technology University, Shenzhen 518118, China

**Keywords:** BioMEMS sensor, continuous monitoring, self-powered sensor, sustainable energy harvesting, AI

## Abstract

Continuous monitoring of environmental and physiological parameters is essential for early diagnostics, real-time decision making, and intelligent system adaptation. Recent advancements in bio-microelectromechanical systems (BioMEMS) sensors have significantly enhanced our ability to track key metrics in real time. However, continuous monitoring demands sustainable energy supply solutions, especially for on-site energy replenishment in areas with limited resources. Artificial intelligence (AI), particularly large language models, offers new avenues for interpreting the vast amounts of data generated by these sensors. Despite this potential, fully integrated systems that combine self-powered BioMEMS sensing with AI-based analytics remain in the early stages of development. This review first examines the evolution of BioMEMS sensors, focusing on advances in sensing materials, micro/nano-scale architectures, and fabrication techniques that enable high sensitivity, flexibility, and biocompatibility for continuous monitoring applications. We then examine recent advances in energy harvesting technologies, such as piezoelectric nanogenerators, triboelectric nanogenerators and moisture electricity generators, which enable self-powered BioMEMS sensors to operate continuously and reducereliance on traditional batteries. Finally, we discuss the role of AI in BioMEMS sensing, particularly in predictive analytics, to analyze continuous monitoring data, identify patterns, trends, and anomalies, and transform this data into actionable insights. This comprehensive analysis aims to provide a roadmap for future continuous BioMEMS sensing, revealing the potential unlocked by combining materials science, energy harvesting, and artificial intelligence.

## 1. Introduction

Bio-microelectromechanical systems (BioMEMS) have attracted increasing attention in recent years, particularly in biological and medical applications. Among BioMEMS devices, continuous sensors have gained prominence for enabling real-time tracking of biomolecular and physiological signals. These sensors offer valuable insights into dynamic changes within biological systems. They support progress in healthcare, biotechnology, environmental monitoring, and fundamental research. Traditional laboratory methods provide only discrete measurements. In contrast, continuous BioMEMS sensors offer uninterrupted data streams that reveal subtle fluctuations and long-term trends. This capability is essential for applications such as long-term monitoring in chronic disease management, for example, in diabetes care. Continuous monitoring is also important in environmental surveillance. It enables the real-time detection of pollutants and toxins, supporting timely intervention to reduce potential harm. In industrial process control, continuous BioMEMS sensors provide real-time feedback on key variables. This helps optimize bioprocesses, improving efficiency and product quality. However, most commercial continuous BioMEMS sensors rely on enzymatic technologies and are limited to detecting a narrow range of targets, such as glucose, lactate, and cortisol. This limitation has motivated the development of alternative approaches, such as affinity-based detection, to broaden the range of detectable analytes. Moreover, continuous monitoring of protein and peptide biomarkers remains challenging due to the lack of suitable enzymes for signal generation. Novel methodologies including single-molecule plasmon-enhanced fluorescence are offering promising avenues for continuous monitoring of diverse biomolecules, including nucleic acids, proteins and peptides.

Despite substantial progress in BioMEMS sensor technology, maintaining continuous operation remains challenging due to the need for sustainable energy sources. Traditional batteries are limited by short lifespan and environmental concerns, especially in remote or resource-limited settings. Researchers have begun exploring self-powered techniques to overcome this limitation. These methods harvest energy from ambient sources and reduce dependence on external power, contributing to sustainability. One promising approach involves piezoelectric nanogenerators (PENGs), which convert mechanical energy from sources like body movement or vibrations into electrical energy. Similarly, triboelectric nanogenerators (TENGs) utilize the triboelectric effect, generating electricity from the contact and separation of different materials. These nanogenerators can be integrated into wearable BioMEMS sensors, allowing continuous operation without relying on conventional batteries. Moisture electricity generators (MEGs) represent another emerging solution. They harness energy from water evaporation or environmental humidity changes. This technology is well suited to high-humidity environments and offers a sustainable energy source for continuous BioMEMS sensing. Furthermore, power management interfaces are essential for self-powered sensors. They convert and regulate harvested energy to maintain reliable sensor operation.

The large volume of data generated by continuous BioMEMS sensors requires advanced analytical methods to extract meaningful insights. Artificial intelligence (AI), particularly machine learning (ML), has become a key enabler of predictive analytics in BioMEMS sensing. Classic AI algorithms like K-nearest neighbors (KNNs) and linear discriminant analysis (LDA) have been successfully applied to analyze sensor data and identify patterns. KNNs classify data points based on their proximity to known data points, while LDA finds linear combinations of features that best separate different classes. These algorithms can classify data, predict future trends, and detect anomalies which provide valuable information for decision making. Advanced AI models continue to expand the scope of predictive analytics in BioMEMS sensing, moving beyond traditional deep neural networks (DNNs) and natural language processing (NLP) approaches. While DNNs are effective at learning complex patterns in data, newer models such as Transformers are changing how researchers interpret signals from BioMEMS sensors. Transformers, originally designed for sequence transduction tasks like language translation, leverage self-attention mechanisms to weigh the importance of different data points in a sequence. This mechanism allows the model to capture long-range dependencies and contextual information which is crucial for understanding the dynamic nature of biological signals. For instance, in continuous glucose monitoring, Transformers can effectively model the interplay between glucose levels, insulin dosage, and meal patterns, leading to more accurate predictions and personalized insights for diabetes management. Furthermore, Graph Neural Networks (GNNs) also show promise for analyzing BioMEMS sensor data with inherent graph structures. GNNs can capture relationships between different sensors or biomolecules, enabling a more holistic understanding of the underlying biological processes. For example, in wearable sensor networks monitoring physiological signals like heart rate, respiration and skin temperature, GNNs can model the interactions between these signals to provide a comprehensive assessment of an individual’s health status. Large Language Models (LLMs), known for natural language understanding and generation, are increasingly applied in BioMEMS-related sensing tasks. LLMs can analyze textual information associated with sensor data, such as patient medical records or environmental reports, to provide a richer context for interpretation. The integration of AI with BioMEMS sensors enables the transformation of raw data into actionable insights. This supports advances in healthcare, personalized diagnostics, and environmental monitoring.

**Figure 1:**
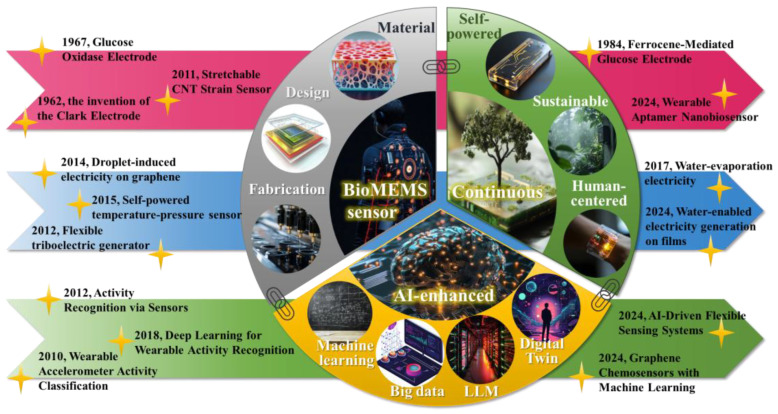
Technological milestones and thematic pillars driving the evolution of intelligent and self-powered BioMEMS sensors.

In this work, we first trace the evolution of continuous BioMEMS sensors through a review of their materials, design principles, and fabrication techniques. Their applications are demonstrated across key areas such as real-time physiological monitoring, noninvasive detection of biomarkers, and wearable sensing platforms. Next, we delve into recent advances in energy harvesting technologies, which address the critical challenge of sustainable power supply for continuous BioMEMS sensors. Finally, we discuss the transformative role of AI in BioMEMS sensing, with a focus on predictive analytics. We examine how AI algorithms, including classic machine learning techniques and state-of-the-art models like Transformers and GNNs, can analyze continuous monitoring data to identify patterns, trends and anomalies. This analysis emphasizes the potential of AI to translate raw sensor data into actionable insights. Integrating advances in BioMEMS sensors, sustainable self-powered energy harvesting, and AI-enhanced analytics has become essential for building next-generation BioMEMS systems (Figure 1). These advancements collectively hold the potential to fundamentally transform the manner in which we monitor and interact with our world.

## 2. BioMEMS Sensors for Continuous Monitoring

### 2.1. Nanomaterials

Nanomaterials have become a key enabler in the development of continuous BioMEMS sensors. Their unique properties—such as high surface area-to-volume ratio, tunable physicochemical characteristics, and exceptional sensitivity—make them ideal for enhancing the performance of micro-scale biological sensing platforms. These materials contribute to improved detection limits, faster response times, and greater selectivity, while also supporting biocompatibility and long-term operational stability. In the context of BioMEMS sensors, nanomaterials can be classified based on their dimensionality. Zero-dimensional (0D) materials, such as nanoparticles and quantum dots, exhibit nanoscale dimensions in all three spatial directions. One-dimensional (1D) materials, including nanotubes and nanowires, extend macroscopically along one axis while maintaining nanoscale cross-sections. Two-dimensional (2D) materials, such as graphene, feature atomic-scale thickness with planar lateral dimensions, offering large surface areas for functionalization and integration with microfabricated sensor structures.

#### 2.1.1. Particles

- Carbon Black

**Figure 2:**
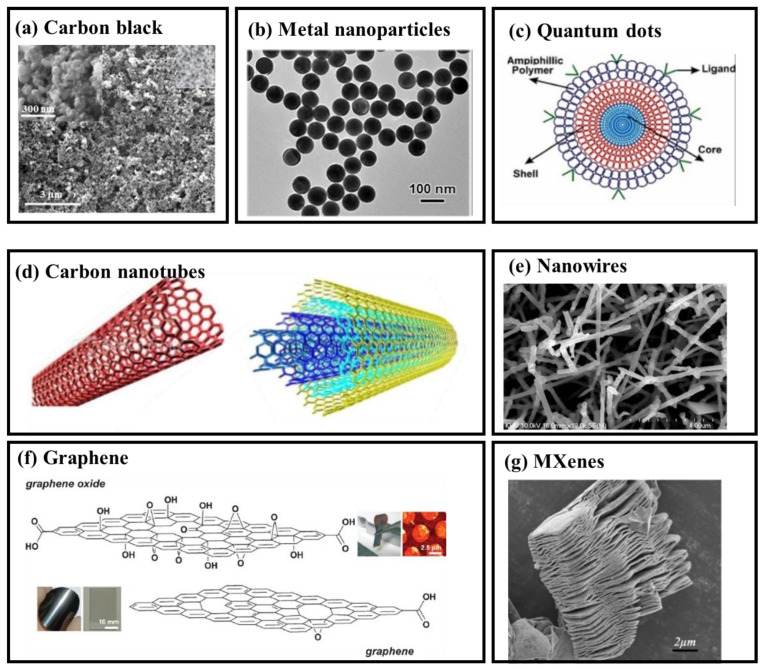
Typical materials and fabrication methods in BioMEMS sensor development. (**a**) Carbon black particles [3]. (**b**) Metal nanoparticles [5]. (**c**) Quantum dots [6]. (**d**) Carbon nanotubes [7]. (**e**) Nanowires [8]. (**f**) Graphene and its derivatives [9]. (**g**) MXenes [10].

Carbon black (CB) is produced from the incomplete combustion of oil products. It mainly consists of carbon and hydrogen, with trace amounts of nitrogen, sulfur, and oxygen [1]. In BioMEMS fabrication, CB particles exhibit nanoscale spherical morphology with diameters ranging from 3.0 to 100 nm (Figure 2a). These particles offer a large specific surface area, excellent electrochemical properties, and ease of functionalization. These characteristics make CB an ideal electrode modifier in BioMEMS sensors, particularly for integration within microfabricated platforms [2]. Functionalization strategies, such as increasing oxygen-containing groups, enhance CB’s wettability and electron transfer capabilities, thereby improving sensor performance. For example, functionalized CB demonstrated a 100-fold increase in performance for catechol detection [3]. In non-enzymatic glucose detection, CB-decorated conductive carbon ink-modified polyimide substrates exhibited selective sensing of glucose in phosphate buffer [4]. CB’s tunable surface chemistry and high conductivity significantly enable seamless integration into miniaturized BioMEMS diagnostic systems.

- Metal Nanoparticles

Metal nanoparticles (MNPs) (Figure 2b), including gold, silver, and copper, are increasingly integrated into BioMEMS sensors owing to their high surface area-to-volume ratio, adjustable size, and superior biocompatibility [11]. Copper nanoparticles electrochemically deposited on platinum electrodes create a biocompatible microenvironment for Mycobacterium tuberculosis DNA detection, achieving a 1 nM limit of detection (LOD) [12]. Additionally, citrate-capped gold nanoparticles (AuNPs) modified with chemically synthesized peptide receptors have been utilized for the detection of COVID-19 antibodies [13]. The functionalization of AuNPs with flavin adenine dinucleotide has also been explored for the selective sensing of dopamine at nanomolar levels, with a LOD of 525 nM [14]. These examples demonstrate the versatility of MNPs in enhancing sensitivity and selectivity across a wide range of biomolecular targets. Their compatibility with microfabrication techniques further supports their application in high-performance, continuous BioMEMS sensing systems.

- Quantum Dots

Quantum dots (QDs), zero-dimensional nanostructured semiconductors (Figure 2c), exhibit unique optical and electronic properties due to their few-nanometer size and atomic composition [15]. QDs’ size, shape, composition, and structure can be tuned for specific wavelengths, making them highly versatile in BioMEMS sensor applications [16]. For example, a cholesterol-detecting BioMEMS sensor was developed using graphene quantum dots (GQDs), cerasome, and cholesterol oxidase. These components were assembled layer by layer on a glassy carbon electrode. The device exhibited electrocatalytic activity over a linear detection range from 16 µM to 6.186 mM, with a detection limit of 5 µM in phosphate buffer [17]. Additionally, a glioma cell BioMEMS sensor was created with sulfur-doped GQDs on gold nanoparticle-decorated carbon nanospheres [18]. These examples highlight the high tunability and sensitivity of QDs, along with their compatibility with microfabricated electrodes and layered assemblies, enabling their seamless integration into high-performance BioMEMS platforms.

#### 2.1.2. Nanotubes, Nanowires and 2-D Materials

- Nanotubes

Carbon nanotubes (CNTs), including single-walled (SWCNTs) and multi-walled (MWCNTs) (Figure 2d), are cylindrical nanomaterials with exceptional mechanical, electrical, and thermal properties [19]. The high aspect ratio, large surface area, and excellent conductivity of MWCNTs enhance BioMEMS sensors. In addition, their chemical stability and biocompatibility promote efficient electron transfer at the microscale. A fentanyl-detecting BioMEMS sensor using MWCNTs achieved a low detection limit of 86 ng/mL in phosphate buffer and urine [20]. Similarly, a glucose sensor with a LOD of 0.2 µM was fabricated using glucose oxidase encapsulated in a paraphenylenediamine polymer matrix on CNTs [21]. Additionally, a hydrogen peroxide BioMEMS sensor using horseradish peroxidase and hydroxyapatite-functionalized CNTs was developed [22]. These studies demonstrate the adaptability of CNTs in building microfabricated sensing interfaces.

- Nanowires

Nanowires are one-dimensional nanostructures (Figure 2e) typically synthesized from semiconducting metal oxides, carbon materials, or metal nanotubes [23]. Compared to their bulk counterparts, they possess superior mechanical strength, thermal stability, chemical responsiveness, and electronic conductivity [24]. These attributes support their integration into BioMEMS systems for high-sensitivity molecular recognition. For instance, Hakim et al. fabricated a poly-silicon nanowire-based BioMEMS sensor that demonstrated the ability to sense the joining capacity of two inflammatory biomarkers with a broad concentration range and good detection sensitivity [25]. In another study, Irrera et al. fabricated a label-free silicon nanowire device for detecting C-reactive protein in human serum [26]. Priolo et al. designed an ultrasensitive genome detection platform using silicon nanowires, highlighting their clinical relevance in personalized diagnostics [27].

Nanotubes and nanowires continue to propel the development of BioMEMS sensors by offering electrically conductive, geometrically tunable, and biocompatible structures that enhance sensitivity, selectivity, and long-term stability.

- Graphene

Graphene and its derivatives, including graphene oxide (GO) and reduced graphene oxide (Figure 2f) [28], are two-dimensional materials with excellent potential for BioMEMS sensor applications. Their hexagonal lattice structure provides exceptional mechanical strength, electrical conductivity, and chemical stability. These properties contribute to improved sensitivity and selectivity in electrochemical BioMEMS systems [29]. Parlak et al. (2013) immobilized cholesterol oxidase and esterase on graphene functionalized with anionic surfactants and gold nanoparticles, achieving detection limits of 25 nM for hydrogen peroxide (H_2_O_2_) and 50 nM for cholesterol [30]. Similarly, Pan et al. fabricated a BioMEMS sensor for bisphenol A using a graphene-gold nanoparticle nanocomposite to immobilize tyrosinase and chitosan, with a linear detection range of 2.5 nM to 3 µM [31]. Poletti et al. developed a sweat sensor based on GO for glucose and lactate detection, with limits of quantification of 32 nM and 68 nM, respectively [32]. These studies underscore the suitability of graphene-based materials for high-performance, miniaturized biosensing platforms.

- MXenes

MXenes are a family of two-dimensional transition metal carbides, nitrides, and carbonitrides derived from MAX phases through selective etching (Figure 2g) [33]. These materials possess high electrical conductivity, large surface area, and inherent hydrophilicity, which support efficient biomolecule immobilization and signal transduction in BioMEMS sensors. Wang et al. [34] developed a mediator-free H_2_O_2_ sensor by encapsulating hemoglobin within Ti_3_C_2_ MXene. In environmental monitoring, a phenol sensor using 2D transition metal carbide MXene with immobilized tyrosinase was reported [35]. For agricultural applications, Zhao et al. employed Au-Pd nanoparticles on MXene nanosheets to detect paraoxon, achieving low-concentration detection in real samples like cucumber and pear, highlighting MXenes’ versatility across various fields [36]. Compared to one-dimensional or particle-based materials, two-dimensional structures like graphene and MXenes offer larger active areas and planar configurations. These features promote high adsorption capacity, enhanced electron transfer, and efficient integration with microfabricated substrates—making them well suited for continuous BioMEMS sensing platforms.

The performance of continuous BioMEMS sensors is closely linked to the dimensionality, conductivity, functionalization potential, and biocompatibility of nanomaterials. Zero-dimensional particles offer excellent dispersibility and ease of modification but may suffer from limited interconnectivity. One-dimensional structures such as nanotubes and nanowires enable directional conductivity and efficient signal transduction, suitable for elongated sensing interfaces. Two-dimensional materials provide large surface areas and planar compatibility with microfabrication, making them advantageous for layered integration and high-density sensor arrays. However, challenges remain in achieving long-term stability, reproducible surface modification, and scalable manufacturing. Table 1 summarizes the representative features, advantages, and limitations of each nanomaterial class for BioMEMS applications.

### 2.2. Design and Fabrication

#### 2.2.1. Design

BioMEMS sensor design has evolved from the conventional architecture—consisting of a biorecognition element, a transducer, and a signal processor—into highly integrated microsystems that combine mechanical, electrical, and biochemical domains. These platforms are tailored for continuous, real-time operation in dynamic biological environments. Continuous BioMEMS sensors must overcome key design challenges such as biocompatibility, long-term material stability, and microscale system integration. For implantable devices, designs must employ biocompatible and, in some cases, biodegradable materials to ensure safe operation over extended periods. A representative example is the Continuous Glucose Monitor (CGM), a widely used wearable microfabricated sensor often referenced in BioMEMS contexts. However, the continuous monitoring of more complex or low-abundance analytes remains difficult, motivating the development of reversible affinity-based sensing mechanisms and self-regenerating interfaces. Recent trends also emphasize AI-driven co-optimization of structure and function in BioMEMS design. This includes simulation-informed material selection, design-space exploration, and data-driven adaptive calibration. By integrating miniaturized structures, smart materials, and embedded electronics, modern BioMEMS platforms achieve improved accuracy, reduced invasiveness, and broader utility in clinical diagnostics, environmental monitoring, and industrial sensing.

#### 2.2.2. Fabrication

Early BioMEMS sensors were fabricated using traditional methods such as physical adsorption or covalent attachment of biorecognition elements onto transducer surfaces. Although effective, these approaches often lacked stability and reproducibility over time. The advent of microfabrication and nanofabrication technologies, including photolithography, soft lithography, and etching techniques like DRIE, has transformed BioMEMS fabrication. These processes enable the creation of miniaturized, integrated, and multi-functional microsystems with high precision and throughput. Microfluidic architectures now play a central role in fabricating lab-on-a-chip BioMEMS platforms, offering precise fluid control for point-of-care diagnostics. These systems are especially suited for wearable and implantable devices where continuous, real-time monitoring is essential. The incorporation of advanced materials—such as graphene, MXenes, and flexible substrates like PDMS and polyimide—further enhances device biocompatibility, conformability, and mechanical durability. Several fabrication challenges must be addressed. Maintaining the functional stability of immobilized biorecognition elements is crucial, particularly for long-term in vivo use. Strategies such as encapsulation within hydrogel matrices or covalent crosslinking with silane/thiol chemistry can improve robustness. Additionally, minimizing non-specific binding is necessary to preserve sensing specificity. This is often achieved using selective membranes, microfluidic separation, or surface chemistry modification. As BioMEMS sensors advance toward real-world deployment, fabrication strategies increasingly focus on scalability, robustness, and CMOS-compatibility. AI-assisted fabrication workflows are also emerging, enabling defect detection, real-time process monitoring, and predictive control for yield-aware sensor manufacturing.

### 2.3. Monitoring Target

Continuous BioMEMS sensors are designed to detect specific biochemical targets in real time, offering a pathway toward dynamic health and environmental monitoring. These analytes range from small metabolites like glucose and lactate to more complex biomarkers such as proteins and nucleic acids. The design of BioMEMS platforms must be tailored to the physicochemical characteristics of the target, ensuring sensitivity, specificity, and long-term stability under continuous operation.

#### 2.3.1. Glucose

**Figure 3:**
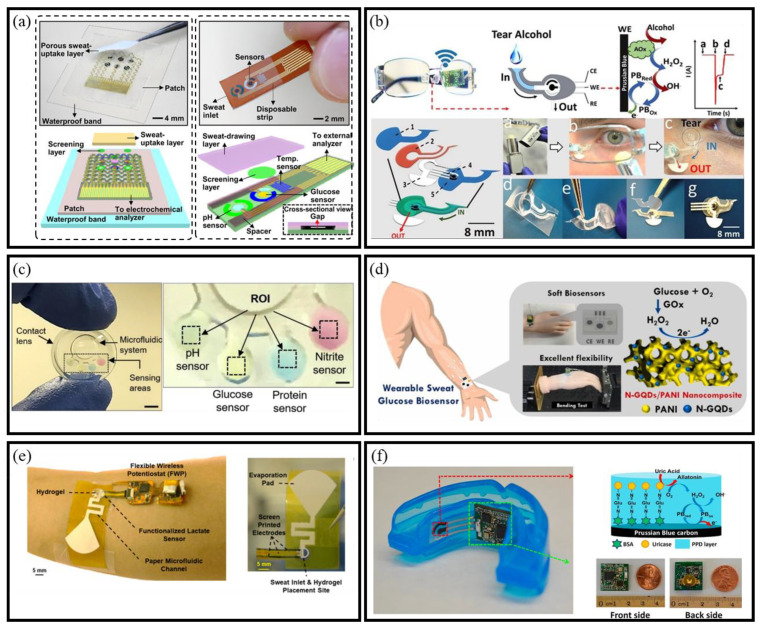
Applications of BioMEMS sensors in Point-of-Care Testing. (**a**) Wearable sweat-based BioMEMS glucose sensor [37]. (**b**) Wearable BioMEMS sensor in glasses for tear glucose monitoring [38]. (**c**) Microfluidic contact lenses for in situ tear sensing [40]. (**d**) Flexible wearable BioMEMS glucose sensor using N-GQDs anchored PANI matrix [41]. (**e**) Wearable BioMEMS platform for continuous sweat lactate monitoring, reprinted with permission from [42]. Copyright 2022 American Chemical Society. (**f**) Mouthguard BioMEMS sensor for salivary uric acid monitoring [43].

Continuous glucose monitoring remains a primary application of BioMEMS sensors, especially for diabetes management. Many systems employ enzyme-based electrochemical detection using glucose oxidase, coupled with microfluidic substrates or flexible electronics to enable continuous and non-invasive operation. A notable example of this innovation is the wearable sweat-based BioMEMS glucose sensor developed by Lee et al., which not only detects glucose levels but also facilitates drug delivery in response to fluctuations in glucose concentration (Figure 3a) [37]. Similarly, Sempionatto et al. developed a wearable BioMEMS sensor embedded in glasses for non-invasive tear glucose monitoring [38]. This device overcomes the limitations of direct eye contact systems, enabling real-time analysis of health biomarkers, including alcohol levels (Figure 3b). Further exemplifying this trend, Jia et al. created GO-coated microfluidic paper-based analytical device integrated with a smartphone-based colorimetric system for oral fluid glucose quantification [39]. Another pioneering approach comes from microfluidic contact lenses [40], designed for in situ sensing of glucose, pH, protein, and nitrite ions in tears, integrating four BioMEMS sensors that utilize a colorimetric method and are paired with a smartphone-MATLAB algorithm (Figure 3c). Additionally, Lin et al. developed a flexible wearable BioMEMS glucose sensor with high detection accuracy [41], utilizing an N-doped graphene quantum dots anchored polyaniline matrix (Figure 3d). These examples demonstrate the transition from traditional glucose tests to continuous, intelligent, and minimally invasive BioMEMS solutions.

#### 2.3.2. Lactate Acid

Lactate is a key metabolic biomarker produced during anaerobic respiration in muscle tissues under hypoxic conditions. Its accumulation correlates with muscle fatigue and soreness, making it a valuable target for physiological monitoring [44,45]. Since lactate concentration in sweat is typically 2–10 times higher than in blood, sweat has emerged as a suitable medium for non-invasive detection. Given the heightened interest in noninvasive detection methods, numerous lactate BioMEMS sensors are being developed using sweat as a sample [46]. Saha et al. developed a wearable BioMEMS platform that integrates with osmotic sweat extraction to continuously monitor sweat lactate (Figure 3e) [42]. This study employed screen-printed electrodes (SPEs) with immobilized LOx at the sweat collection site on the skin for dynamic assessment of lactate levels. These advancements underscore the potential of BioMEMS lactate sensors in enabling real-time, non-invasive monitoring of muscle fatigue, with promising applications in sports performance optimization, rehabilitation, and physical exertion assessment.

#### 2.3.3. Uric Acid

Uric acid (UA) is a critical indicator of various health conditions, with levels typically ranging from 240 to 520 μmol/L in healthy individuals [47]. Elevated UA levels can lead to diseases such as gout [48] and hyperuricemia [49]. Monitoring UA is thus essential for early diagnosis and treatment. Saliva-based BioMEMS sensors provide a non-invasive alternative to conventional blood tests for UA detection. Shi et al. developed an enzymatic BioMEMS platform that used screen-printed electrodes modified with MWCNTs and uricase to measure UA levels in saliva [50]. Another platform for sensing UA from saliva involves the use of a mouthguard sensor. Kim et al. created a mouthguard BioMEMS sensor for salivary UA monitoring (Figure 3f) [43]. These wearable systems demonstrate the feasibility of real-time, point-of-care uric acid monitoring, offering new possibilities for early diagnosis and management of uric acid–related conditions.

To conclude, recent advances in nanomaterials, fabrication techniques, and system-level architectures have greatly expanded the capabilities of BioMEMS sensors for continuous monitoring. These innovations collectively enable miniaturized, flexible, and high-performance platforms that can be tailored for diverse biomedical and environmental applications. However, as BioMEMS devices become increasingly autonomous and deployed in resource-constrained settings, the reliance on conventional batteries poses a major limitation. This highlights the urgent need for sustainable, miniaturized energy harvesting strategies.

## 3. Sustainable Power Supply for BioMEMS Sensor

Despite significant advances in environmental and biomedical sensing, the sustainable powering of continuous BioMEMS sensors remains a critical challenge. The reliance on conventional batteries introduces limitations related to size, flexibility, biocompatibility, and operational lifetime, particularly in wearable or implantable systems. Self-powered BioMEMS sensors, which harvest energy from ambient sources such as mechanical motion, surface contact, or moisture gradients, offer a promising alternative. Integration of energy harvesting modules—such as PENGs, TENGs, and MEGs—can eliminate the need for bulky external power supplies. These energy solutions are increasingly being adapted for seamless incorporation into MEMS-scale architectures, supporting autonomous, long-term operation.

### 3.1. Piezoelectric Nanogenerators

A piezoelectric nanogenerators (PENGs) is a device that converts mechanical energy at the nanoscale into electrical energy by exploiting the piezoelectric effect [51]. This reversible effect allows PENGs to efficiently harvest energy from mechanical deformations, making them ideal for small-scale energy conversion applications. In the context of BioMEMS, PENGs have demonstrated excellent compatibility with wearable substrates and microscale sensor layouts. Their integration supports continuous energy supply from physiological motion (e.g., limb movement, respiration), enabling real-time sensing without external power sources. The materials commonly used—such as ZnO nanowires, PVDF-based composites, or doped ceramics—offer high energy conversion efficiency and are amenable to microfabrication techniques.

#### 3.1.1. Mechanism of PENGs

**Figure 4:**
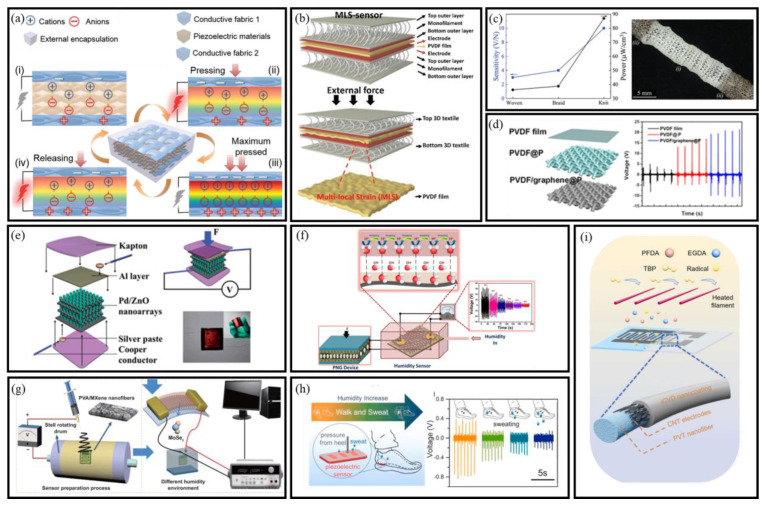
PENGs’ Mechanism, architectures and applications in self-powered BioMEMS sensors. (**a**) Schematic of the piezoelectric charge generation process: (i) charge equilibrium; (ii) dipole formation under compression; (iii) peak polarization at maximum strain; (iv) charge rebalance upon release [53]. (**b**) Nanofiber Membrane-Based PENGs: (top) multi-local strain sensor (MLS-sensor) structure and (bottom)deformation of 3D textile and multi-local strain in PVDF film when external force is given. [55]. (**c**) Yarn-based flexible PENGs: (left) comparison of sensitivity and power output of textile-based energy generators, (right) wearable generator using seamless electrodes; (i) knitted PVDF/BT10 fiber, (ii) silver-coated nylon yarn as integrated electrode. [56]. (**d**) Fabric-based PENGs: (left) schematic comparison of PVDF film, PVDF@P, and PVDF/graphene@P structures; (right) corresponding voltage output of the three configurations. Reprinted with permission from [57]. Copyright 2018 American Chemical Society. (**e**) Self-powered active gas sensor based on Pd/ZnO nanoarray PENGs: (left) schematic of device assembly; (upper right) final structure illustrating piezoelectric output variation with ethanol concentration; (lower right) optical image showing device flexibility. [58]. (**f**) Self-powered piezoelectric biopolymer humidity sensors: schematic of humidity sensing mechanism and corresponding voltage response under varying relative humidity conditions. Reprinted with permission from [59]. Copyright 2018 American Chemical Society. (**g**) Self-powered flexible humidity sensor based on electrospun PVA/MXene nanofibers: schematic of sensor fabrication and humidity sensing measurement platform. [60]. (**h**) Self-powered pressure sensor based on electrospun PVDF-TrFE/MXene film: (left) schematic of heel-mounted sensor and (right) corresponding voltage response under sweat-induced pressure [61]. (**i**) Robust superhydrophobic antibacterial self-powered PENG sensor [62].

The fundamental principle of electricity generation in piezoelectric materials involves breaking central symmetry in their crystal structure under an external force, creating a piezopotential [52]. Figure 4 illustrates this process and the electrical output characteristics during a compression and decompression cycle [53]. Initially, the charge centers of cations and anions coincide, resulting in no observable polarization within the piezoelectric material (Figure 4a-i). Upon applying a pressing force, the deformation of the piezofabric generates a negative strain and reduces the volume. This separation of charge centers forms electrical dipoles, causing changes in the dipole moments and creating a piezopotential between the electrodes (Figure 4a-ii). Consequently, mechanical energy is converted into electrical energy. When the conductive fabric electrodes make full contact, the system reaches its maximum pressed state, exhibiting the highest polarization density (Figure 4a-iii). Upon releasing the external force, electrons return to rebalance the charge induced by the strain release, as shown in the short-circuit condition (Figure 4a-iv). Therefore, when the piezopotential is continuously modified by dynamic strain, a steady pulse current is generated through the external circuit [54]. In the context of BioMEMS sensors, this mechanism enables real-time energy harvesting from physiological motion or environmental stimuli. The compatibility of PENGs with flexible substrates and MEMS-scale integration underscores their potential in autonomous microsystems.

#### 3.1.2. Architectures of PENGs

This section outlines representative PENG configurations—including nanofiber membrane, yarn-based, and fabric-integrated structures—designed to enhance energy harvesting performance in flexible BioMEMS platforms. These structures, utilizing materials like PVDF, graphene, and composites, are integral to enhancing the piezoelectric performance, sensitivity, and durability of wearable energy-harvesting devices.

- Nanofiber Membrane-Based PENGs

Nanofiber membrane-based PENGs are typically constructed in a sandwich-like structure, comprising two flexible electrodes with a piezoelectric nanofiber membrane in between. This configuration enhances sensor durability and signal transmission stability. Upon external stimulation, the piezoelectric layer generates opposite charges, producing a voltage proportional to the applied mechanical stress. Yang et al. designed a 3D-interlocked PVDF/ZnO membrane to optimize stress distribution [63], while Shahzad et al. embedded GO in P (VDF-TrFE) fibers, enhancing charge generation [64]. Ahn et al. further integrated pre-strained 3D textiles (Figure 4b) into wearable sensors, transforming pressure absorption into active stress transmission, enabling pressure-to-stress conversion for breathable biomechanical sensors [55]. These membrane-type PENGs exhibit scalable design and mechanical adaptability, supporting integration into skin-interfaced or implantable BioMEMS sensors for continuous biomechanical monitoring.

- Yarn-Based Flexible PENGs

Yarns with small structural elements provide e-textiles with diverse functionalities, including self-cleaning, piezoelectricity, and enhanced strength. To create wearable devices, soft and flexible yarns with high surface area and sensitivity can be seamlessly incorporated into fabrics through methods such as weaving, knitting, sewing, or embroidery. These yarns are capable of directly converting mechanical energy into electrical energy. High-aspect-ratio piezoelectric yarns offer an attractive route for fabric-based energy harvesting systems, particularly those embedded in wearable self-powered BioMEMS sensors. Mokhtari et al. integrated melt-spun PVDF/BT_10_ yarns with 98% β-phase, achieving 4 V voltage and 87 µW cm^−3^ power (Figure 4c). Seamless silver-coated nylon electrodes enable sixfold faster 10 µF capacitor charging, while tunable hydraulic/pneumatic sensitivity highlights multifunctional e-textile energy-sensor synergy [56].

- Fabric-Based PENGs

The output of PENGs made from a single yarn is limited due to the small active area. To enhance the piezoelectric output, one effective approach is to weave multiple yarns together using textile technology, thereby maximizing the active working area. As a result, numerous researchers have focused on developing PENGs from textiles, leading to significant advancements in the field. Huang et al. fabricated fabric-based PENGs by phase-separation-assisted coating of PVDF/graphene composites onto polyester fabric (Figure 4d) [57]. The structured fabric exhibited 34 V/N sensitivity, enabling micro-force detection (voice/body motions), highlighting textile-specific β-phase enhancement for piezoelectric optimization. The textile-based PENGs not only offer enhanced power output but also enable system-level expansion via weaving-based scalability. These features are highly desirable for next-generation self-powered BioMEMS sensor arrays.

#### 3.1.3. PENG-Based Self-Powered BioMEMS Sensors

PENGs have gained attention as a robust solution for self-powered sensing. By converting mechanical deformation into electrical signals, PENGs support autonomous operation in environmental, industrial, and biomedical systems. Their compact form and low-power output make them suitable for integration into miniaturized platforms. In gas sensing, Lin et al. pioneered a flexible Pd/ZnO nanowire array sensor on Ti foil, where mechanical finger bending generated piezoelectric potentials to simultaneously power the device and modulate its ethanol sensitivity at room temperature (Figure 4e) [58]. Building on heterostructure design, Qu et al. demonstrated high H_2_S sensitivity and selectivity using a NiO/ZnO PN-junction NW NG as a self-powered gas sensor [65]. For humidity monitoring, Vivekananthan et al. fabricated a self-powered piezoelectric biopolymer humidity sensors using collagen nanofibrils on cotton cloth (Figure 4f) [59]. The device demonstrated self-powered sensing capabilities by connecting with a collagen PENG device. Furthermore, Wang et al. reported a self-powered flexible humidity sensor using electrospun PVA/MXene nanofibers film and monolayer MoSe_2_ piezoelectric nanogenerator on PET substrate (Figure 4g) [60]. These innovations underscore PENGs’ adaptability in addressing critical industrial and agricultural monitoring challenges.

Structural and material optimization enables PENGs to overcome traditional rigidity limitations for wearable integration. Wang et al. reported a piezoelectric film pressure sensor fabricated from electrospun PVDF-TrFE/MXene nanofiber mats (Figure 4h) [61]. The composite film demonstrated significantly enhanced output voltage compared to neat PVDF-TrFE. Li et al. developed a self-powered, porous, flexible, hydrophobic, and breathable PVDF nanofiber membrane as a tactile sensor for human body motion detection [66]. Further enhancing clinical applicability, Su et al. reported a robust superhydrophobic antibacterial self-powered PENG-based sensor for human behavior monitoring [62]. The sensor featured a flexible PVDF-TrFE nanofiber membrane with carbon nanotube electrodes and an iCVD hydrophobic nanocoating (Figure 4i).

Together, these examples demonstrate the potential of PENG-based sensors to support self-powered BioMEMS systems. Their ability to combine high-performance sensing with mechanical compliance offers promising avenues for healthcare monitoring, human–machine interfaces, and autonomous environmental sensing.

### 3.2. Triboelectric Nanogenerators

The triboelectric effect, common in daily life, generates and transfers charges through contact or friction between materials. In 2012, Professor Zhong Lin Wang’s research group pioneered the Triboelectric Nanogenerators (TENGs), combining triboelectrification with electrostatic induction [67]. This innovation opened new possibilities for mechanical energy harvesting in self-powered microsystems

#### 3.2.1. Mechanisms of TENGs

**Figure 5:**
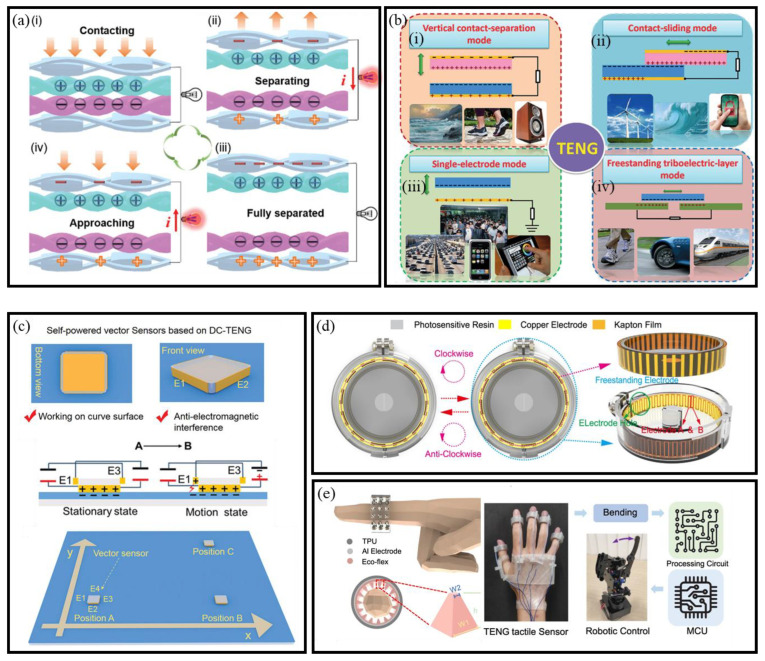
TENGs’ mechanism, working modes and applications in self-powered BioMEMS sensors. (**a**) Basic working mechanism of TENGs: (i) contact electrification under pressure; (ii) separation-induced potential difference and electron flow; (iii) maximum potential at full separation; (iv) reapproach of triboelectric layers under external force. [53]. (**b**) Four fundamental modes of TENGs: vertical contact separation, lateral sliding, single-electrode, and freestanding triboelectric-layer modes [68]. (**c**) Self-powered motion vector sensor based on DC-TENGs: (top) schematic structure of the DC-TENG-based motion vector sensor and comparison with conventional AC-TENG; (middle) planar displacement sensing mechanism; (bottom) DC-TENG design for motion quantification [69]. (**d**) Badge-reel-like stretch sensor based on grating-structured TENGs: top view illustrates clockwise and anticlockwise rotation; right side shows 3D structural details of the device [70]. (**e**) TENG-based tactile sensor integrated into a robotic manipulator: (left) structural design of the tactile sensor; (right) schematic of the collaborative robotic operation system [71].

The triboelectric effect occurs when materials acquire charge through frictional contact with other materials of different electronegativities [68]. TENGs utilize this process to convert kinetic energy into electricity, offering a viable power source for wearable and BioMEMS-based sensors (Figure 5a) [53]. Initially, two distinct materials come into contact under external force, generating equal but opposite charges on their surfaces through the process of contact electrification (Figure 5a-i). At this stage, due to the overlap of the charge distributions in the same plane, the system is in an electrically neutral state. Upon removing the external force, the materials separate, creating a potential difference between the two electrodes. If the electrodes are short-circuited, this potential difference drives electrons to flow from the lower to the upper electrode, resulting in a transient pulse current (Figure 5a-ii). When the external force is applied again (Figure 5a-iii), the two triboelectric materials approach each other (Figure 5a-iv), causing the potential of the upper electrode to decrease relative to that of the lower electrode. This repeated contact–separation cycle generates a periodic alternating current, enabling the TENG to continuously convert mechanical motion into electrical output. The simplicity of TENG operation, along with its scalability and compatibility with soft materials, makes it particularly promising for self-powered wearable devices and BioMEMS sensors.

#### 3.2.2. Working Modes of TENGs

TENGs operate in four primary modes, each utilizing distinct mechanisms for converting mechanical energy into electrical energy. These modes are vertical contact separation, contact-sliding, single-electrode, and freestanding triboelectric-layer modes (Figure 5b) [68].

- Vertical Contact Separation Mode

In the vertical contact separation mode of TENGs, two dissimilar dielectric films are arranged face-to-face, with electrodes deposited on the top and bottom surfaces [72] (Figure 5b-i). The operation begins with an external force prompting contact between the films, triggering contact electrification and creating oppositely charged surfaces. Upon force removal, film separation induces a potential drop across the electrodes. Connecting the electrodes to a load allows electrons to flow from one to the other, neutralizing the potential difference and rebalancing the electrostatic field. When the films recontact, the triboelectric potential is nullified, and electrons revert to their initial positions. This cyclic charge accumulation and discharge underpins the fundamental operation of vertical contact separation mode TENGs.

- Contact-sliding Mode

The structure of the contact-sliding mode TENGs is similar to that of the vertical contact separation mode. In this configuration, when two dielectric films come into contact, a relative lateral sliding motion along the surface induces triboelectric charges on both surfaces (Figure 5b-ii) [73]. This sliding motion generates a lateral polarization in the direction of movement, which drives electrons on the top and bottom electrodes to flow, thereby neutralizing the electric field created by the triboelectric charges. A periodic sliding motion, alternating between separation and closure, produces an alternating current (AC) output. The sliding motion in this mode can take various forms, such as planar motion, cylindrical rotation [74], or disk rotation [75]. Theoretical studies have been conducted to better understand the fundamental operation of this mode, as well as the grating-structured TENGs [76].

- Single-Electrode Mode

The single-electrode mode is designed for scenarios where the TENGs’ components cannot be electrically connected to a load, such as when the object partaking in the energy harvesting process is mobile. For example, when a human walks on a floor, the object (in this case, the human body) moves without direct electrical contact with the load. In this mode, one of the electrodes is grounded, while the other electrode remains in proximity to the object (Figure 5b-iii). When the size of the TENGs is finite, the movement of the top object relative to the bottom one alters the local electrical field distribution. This change induces electron exchanges between the bottom electrode and the ground, thus maintaining the potential variation in the electrode.

- Freestanding Triboelectric-Layer Mode

In the freestanding triboelectric-layer mode, a freely moving dielectric layer interacts with two fixed electrodes (Figure 5b-iv), facilitating energy generation, primarily through rotational motion [77]. The symmetric electrodes, positioned beneath the dielectric layer, induce an asymmetric charge distribution as the object moves, driving electron flow to restore electrical equilibrium. This mode is particularly efficient when the dielectric layer is substituted with rolling rods, which reduces energy consumption and enhances durability [78]. In practical applications, such as walking, objects like shoes accumulate static charges through contact with air, with the charges remaining on the surface without continuous friction. This mode has demonstrated effective energy harvesting from human movement and vehicles, underscoring its potential for capturing energy from freely moving objects [79].

These four operational modes offer diverse options for designing flexible and self-powered BioMEMS sensor systems. Their adaptability to different forms of mechanical motion makes TENGs well suited for integration into wearable, environmental, and BioMEMS platforms.

#### 3.2.3. TENG-Based Self-Powered BioMEMS Sensors

TENGs offer a unique advantage in harvesting ambient mechanical energy, enabling the development of self-powered BioMEMS sensors for continuous monitoring across a wide range of applications.

For motion tracking, Yin et al. introduced a vector triboelectric sensor based on DC-TENG architecture, capable of monitoring displacement, velocity, and acceleration simultaneously. The device also showed strong anti-interference performance, making it suitable for complex dynamic environments (Figure 5c) [69]. Extending this concept, Li et al. developed a grating-structured TENG stretch sensor resembling a badge reel, effectively recording joint kinematics and spinal deformations in real time (Figure 5d) [70]. Further advancing wearable motion sensing, Deng et al. constructed a fully stretchable TENG with micro-pyramidal PDMS and TPU nanofiber films, exhibiting high output performance for continuous monitoring of force, frequency, and bending in personal activities [80].

Concurrently, chemical and environmental monitoring has been revolutionized through TENGs’ inherent sensitivity to interfacial reactions. Gao et al. demonstrated a dual-electrode urea BioMEMS sensor utilizing enzyme-catalyzed reactions, achieving ultrahigh sensitivity (4 μM detection limit) for crop health assessment with robust anti-interference against fertilizers [81]. Similarly, Chatterjee et al. leveraged solid–liquid contact electrification between TiO_2_ nanosheets and organic solvents, developing a TENG-based catechin detector with voltage outputs proportional to analyte concentration [82], thereby bridging agricultural and food safety applications. In soft robotics, Sun et al. developed a TENG-based tactile sensor for continuous bending detection, enabling accurate measurement of bending angles independent of speed through voltage integration [71]. Integrated into a robotic manipulator, it demonstrated fast response, high resolution, and simultaneous multi-finger control (Figure 5e). Notably, TENGs have also been applied in non-biomedical scenarios. Hou et al. developed a TENG-based system for trans-media motion monitoring and digital twin replication, demonstrating the technology’s robustness and adaptability in complex dynamic environments [83].

Collectively, these innovations illustrate the versatility of TENGs as a self-powered platform for intelligent BioMEMS sensors, spanning applications in biomechanics, agriculture, food quality control, and human–machine interfaces.

### 3.3. Moisture Electricity Generators

Water plays a crucial role in global energy dynamics, covering 71% of the Earth’s surface and absorbing approximately 35% of incident solar energy [84]. Recent advancements in nanomaterials have enabled the development of moisture electricity generators (MEGs) [85], which can generate power from moisture without specific environmental conditions. These self-powered systems are well-suited for flexible BioMEMS platforms, especially in resource-limited or off-grid settings.

#### 3.3.1. Mechanism of MEGs

- Ion Diffusion Mechanism

**Figure 6:**
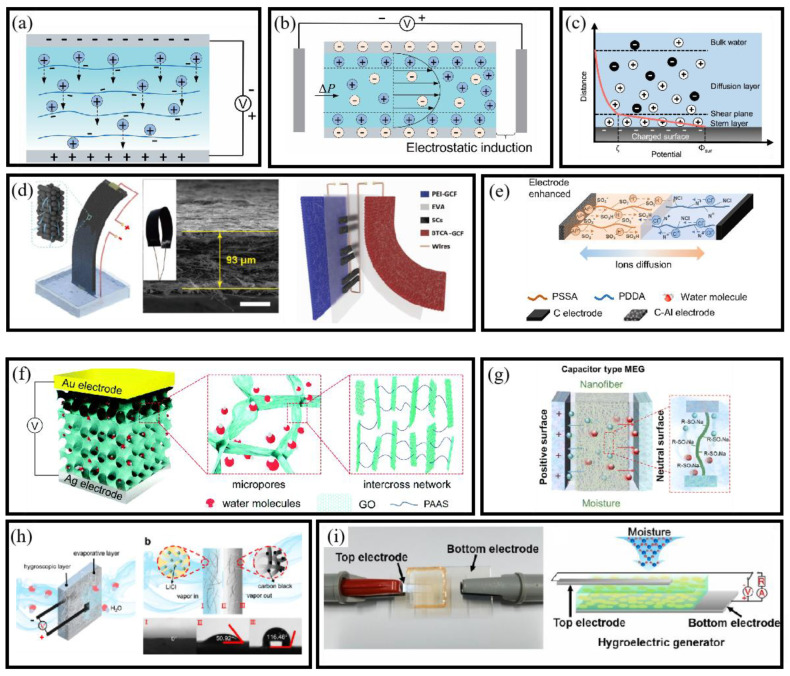
Mechanisms and structural designs of MEGs. (**a**) Ion diffusion mechanism involving functional groups and ion concentration gradients [87]. (**b**) Streaming potential mechanism with water flow through nanochannels [87]. (**c**) EDL formation at water–solid interfaces [88]. (**d**) Planar structure of a flexible carbon nanoparticle film-based nanogenerator [89]. (**e**) Fully printed planar MEGs [90]. (**f**) Sandwich-structured direct-current MEG using porous graphene oxide [91]. (**g**) Sandwich-structured MEG based on EDL capacitor principles [92]. (**h**) Self-sustained electricity generator with a heterogeneous design [93]. (**i**) Asymmetrical microbial whole-cell HEG system for sustainable power generation [94].

The ion diffusion mechanism in MEGs involves the adsorption of water molecules onto functional groups on a material’s surface, inducing ionization and releasing cations and anions [86]. This process creates an ion concentration gradient, generating a potential difference that drives electric current through an external circuit (Figure 6a). Hydrophilic functional groups such as hydroxyl (–OH), carboxyl (–COOH), and sulfonic acid (–SO_3_H) form hydrogen bonds with water and promote ion release: –COOH and –SO_3_H groups can dissociate H^+^ into solution (leaving –COO^−^/–SO_3_^−^), while –OH groups can enhance local water autoprotolysis, increasing H_3_O^+^ and OH^−^ concentrations. Optimizing the mechanism involves engineering ion gradients through functional group distribution and manipulating relative humidity. High humidity enhances water absorption and ion release, strengthening the concentration gradient and improving current output.

- Streaming Potential Mechanism

The streaming potential mechanism refers to the electromotive force generated when water molecules interact with solid surfaces within nanochannels, driven by the electric double layer (EDL) effect [95]. Hygroscopic materials, upon absorbing moisture, create an internal gradient that drives water through nanochannels, generating a directional flow (Figure 6b). As water moves through these channels, it carries ions and molecules with it. In nanoscale pores, water molecules are attracted to charged surfaces, forming an electrical double layer at the water–solid interface (Figure 6c). The migration of these ions generates an electrostatic potential gradient, and the flow of water induces counterion movement, creating an electric field and resulting in streaming potential.

These mechanisms enable the direct conversion of ambient humidity into usable electrical signals, without requiring mechanical components or external stimuli. Their intrinsic compatibility with soft materials and low-power electronics makes them particularly well-suited for integration into flexible BioMEMS platforms.

#### 3.3.2. Structure of MEGs

MEGs consist of electrodes and a hygroscopic active layer. The electrodes extract and transfer charge, while the active layer generates an ion gradient during water absorption. Depending on the active layer and electrode design, MEGs are classified into four types: planar, sandwich, heterogeneous, and asymmetrical structures. Each configuration offers distinct advantages in terms of output stability, structural adaptability, and integration potential with BioMEMS sensing platforms.

- Planar Structure

The planar structure features parallel electrodes and an active layer with pre-designed ion concentration gradients, relying on environmental humidity gradients. The device, partially immersed in deionized water, utilizes capillary action to draw water upward through nanochannels [96]. As water evaporates from the exposed surface, continuous capillary flow enables sustained electricity generation. Li et al. [89] developed a flexible carbon nanoparticle film-based nanogenerator integrated with supercapacitors for powering practical devices (Figure 6d). Similarly, He et al. [90] presented a fully printed planar moisture-enabled electric generator (Figure 6e). These examples highlight the mechanical simplicity and integration potential of planar MEGs in compact sensor systems.

- Sandwich Structure

The sandwich structure comprises stacked electrodes and an active layer with pre-established ion gradients [97]. Incorporating hygroscopic materials like GO or polymer films enables current generation through water adsorption. Increasing film porosity can enhance ion dissociation and boost open-circuit voltage. For instance, Huang et al. developed a direct-current moisture-electric generator using a porous GO and sodium polyacrylate composite (Figure 6f) [91]. Similarly, Sun et al. introduced a moisture-electric generator based on electric double-layer capacitor principles, employing differently charged electrodes and electrolyte-loaded electrospun nanofiber films (Figure 6g) [92]. Sandwich structures offer enhanced encapsulation and are well suited for layered BioMEMS device packaging.

- Heterogeneous Structure

The heterogeneous structure features a bilayer membrane that absorbs moisture and dissociates it into positive and negative ions [98]. The design is enhanced by a multi-layer active layer, which integrates hygroscopic, conductive, and insulating materials, optimizing moisture adsorption, ion migration, and charge transport efficiency [99]. Tan et al. [93] introduced a self-sustained electricity generator with a heterogeneous design, incorporating hygroscopic and evaporative layers made from cellulon papers and additives (Figure 6h). Similarly, Nandakumar et al. developed a DS-PEC system that integrated hygroscopic components with a dye-sensitized semiconductor [100]. This configuration is ideal for multifunctional sensing systems combining humidity and photodetection capabilities.

- Asymmetrical Structure

Unlike sandwich and planar structures, which rely on functional group gradients for rapid dissociation upon water contact, asymmetric structure in MEGs utilizes electrodes with differing moisture permeabilities to create a moisture gradient across the material [101]. This differential permeability facilitates water flow, generating electrostatic potential differences and improving energy conversion efficiency [102]. Li et al. developed asymmetrically patterned CNF/GO composite films as humidity sensors and moisture-driven electricity generators [103]. Ren et al. proposed a microbial whole-cell HEG system (Figure 6i) using a G. sulfurreducens biofilm on ITO glass, generating sustainable power for over 2160 h [94]. These systems exhibit robust long-term operation and are promising for BioMEMS in biomedical and ecological settings.

Together, these architectural strategies provide design flexibility for tailoring MEG performance across multiple application contexts. Whether targeting wearable sensors, implantable biointerfaces, or environmental microsystems, these structures serve as a blueprint for developing next-generation self-powered BioMEMS sensors that are both sustainable and adaptable.

#### 3.3.3. MEG-Based Self-Powered BioMEMS Sensors

**Figure 7:**
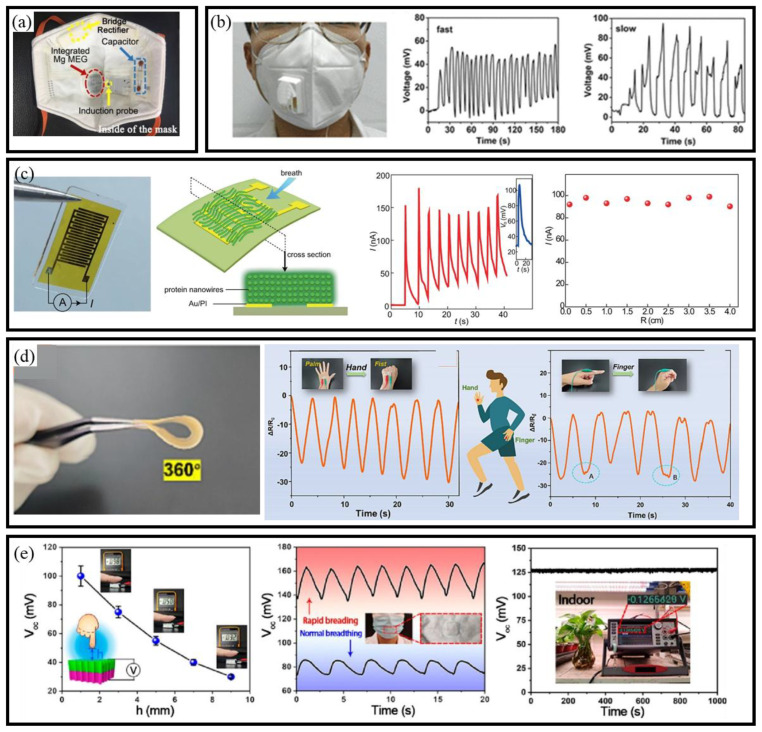
Applications of MEGs in self-powered BioMEMS sensors. (**a**) Hydrophilic polypyrrole nanoarrays integrated MEGs for capturing micropulsed moisture from breathing for physiological monitoring [104]. (**b**) Electrospun cellulose acetate membranes for proton migration-based electricity generation and respiratory pattern detection. Reprinted with permission from [105]. Copyright 2020 American Chemical Society. (**c**) A bio-inspired neuromorphic system based on protein nanowires [106]. (**d**) Asymmetric cellulose acetate honeycomb membrane enhanced with silver nanowires for humidity and motion sensing [107]. (**e**) Self-powered moisture-enabled sensor using a wood-based generator for real-time monitoring of physiological and environmental parameters. Reprinted with permission from [108]. Copyright 2022 American Chemical Society.

Recent developments in MEGs have demonstrated the ability to utilize ambient humidity as a sustainable energy source. Material innovations have played a critical role in advancing MEG-based BioMEMS sensors, enabling real-time operation without external power. For instance, Nan et al. enhanced MEGs’ performance by integrating hydrophilic polypyrrole nanoarrays doped with Mg^2+^ and Al^3+^ [104]. The design also captured and stored micropulsed moisture from human breathing, enabling self-powered, intelligent sensors for physiological monitoring (Figure 7a). Building on the potential of natural materials, Lyu et al. fabricated electrospun cellulose acetate membranes for proton migration and electricity generation [105]. Integrated with silver gauze electrodes, the device could distinguish between different respiratory patterns (Figure 7b).

Further exploring bio-derived materials, Fu et al. developed a bio-inspired neuromorphic system based on protein nanowires. This system coupled MEGs with memristor elements, creating a self-sustaining, adaptive sensing platform powered by environmental moisture (Figure 7c) [106]. In another advancement, Chen et al. fabricated an asymmetric cellulose acetate honeycomb membrane with silver nanowires (Figure 7d), enhancing sensitivity to humidity and human motion, thus advancing porous membranes for moisture-powered sensors [107]. Similarly, Cai et al. [108] introduced a self-powered moisture-enabled sensor using a high-performance wood-based generator (Figure 7e). The sensor tracked finger motion, monitored breath patterns through a mask, and quantified plant transpiration. Furthermore, Lin et al. developed a self-powered MEG using nonwoven fabrics, carbon nanotubes, and liquid alloy [109]. It generates stable power while sensing fluid types by measuring voltage, current, and resistance. Collectively, these advances illustrate the potential of MEG-integrated BioMEMS sensors to operate autonomously in diverse application scenarios, ranging from wearable health monitoring to precision agriculture and environmental diagnostics.

To facilitate a comprehensive understanding of sustainable energy-harvesting strategies, essential performance metrics of PENGs, TENGs, and MEGs are summarized and comparatively analyzed. The evaluation specifically highlights working mechanisms, typical output performance, power density, and representative materials utilized in recent studies. These parameters are systematically detailed in Table 2, enabling readers to clearly identify and compare the relative advantages and limitations inherent to each approach.

## 4. AI-Driven Strategies for Predictive BioMEMS Sensors

Recent advancements in artificial intelligence (AI) and machine learning (ML) have greatly enhanced the predictive capabilities of BioMEMS sensors, enabling smarter and more adaptive microscale platforms [123]. AI algorithms now play a key role in optimizing signal processing, anomaly detection, and decision making within self-powered BioMEMS systems. These capabilities support real-time diagnostics and enable integration with biomedical microrobotics for precise analysis in constrained environments [124].

### 4.1. Machine Learning

**Figure 8:**
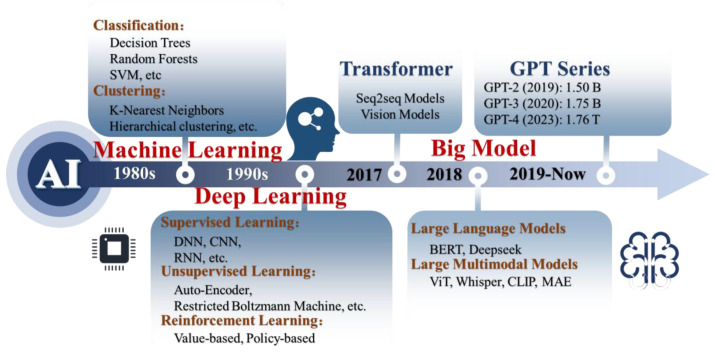
Evolution timeline of artificial intelligence models, illustrating the transition from traditional machine learning, to deep learning approaches, and further toward large-scale models including Transformer architectures, GPT-series, and multimodal foundation models.

ML has played a key role in enhancing signal interpretation and decision making in BioMEMS sensors. Its evolution from classic models to deep learning and large-scale architectures enables it to handle nonlinear, high-dimensional outputs common in biosensing applications (Figure 8).

#### 4.1.1. Classic Machine Learning

- Non-Parametric Models

Non-parametric models offer flexibility in analyzing BioMEMS signals without strict distributional assumptions. Decision trees are a basic method in ML, partitioning datasets into homogeneous subsets by recursively splitting data based on discriminative features [125]. Random Forests enhance decision trees by using multiple trees trained on different data subsets, reducing overfitting through bagging [126]. Naïve Bayes classifiers use Bayes’ theorem assuming feature independence given the class variable [127]. K-Nearest Neighbors assigns class labels based on the majority vote of k nearest training examples [128]. Support Vector Machines (SVMs) are supervised models effective for classification and regression [129].

- Parametric Models

Parametric models provide interpretable mappings from BioMEMS sensor inputs to outcomes when the data is well structured. Parametric models assume a fixed, finite number of parameters, enabling predictions based on a predefined functional form. Logistic Regression calculates event probabilities via a logistic function applied to a linear feature combination, constraining outputs between 0 and 1 [130]. Linear Regression, another fundamental predictive tool, delineates linear dependencies between variables. It posits a linear correlation, estimating coefficients for the optimal fit line to forecast dependent variable values [131]. The Perceptron Model, an early ML algorithm, serves as a linear classifier differentiating two classes based on artificial neuron concepts. Trained through supervised learning, it adjusts weights to reduce prediction errors [132]. While its simplicity aids comprehension, it is confined to linearly separable issues, incapable of tackling nonlinear complexities [133].

Classic machine learning models offer a strong foundation for BioMEMS data analysis. They are easy to implement, require less data, and often provide interpretable results. These methods remain useful for classifying signals, detecting patterns, and supporting decision making in simple or structured biosensing tasks.

#### 4.1.2. Deep Learning

- Supervised Learning

Supervised learning methods are widely applied in BioMEMS platforms to extract interpretable features from labeled sensor data. By minimizing loss functions between predicted and observed outputs, these models enhance classification and regression performance in tasks such as physiological signal monitoring and biochemical concentration tracking. Three predominant models within supervised deep learning include deep neural networks (DNNs), convolutional neural networks (CNNs), and recurrent neural networks (RNNs) [134,135,136]. DNNs consist of multiple layers of neurons that process information hierarchically, with each layer capable of learning increasingly complex features from the data [137]. CNNs are well-suited for extracting spatial features from structured input data, making them particularly effective for BioMEMS sensing applications involving imaging or array-based signal acquisition. Convolutional and pooling layers enhance pattern recognition and dimensionality reduction, improving generalization. RNNs are designed for sequential data and are especially suitable for time-series BioMEMS outputs, such as real-time glucose dynamics, respiration cycles, or heart rate variability. These models retain past information, allowing them to capture temporal dependencies critical for continuous monitoring tasks.

- Unsupervised Learning

Unsupervised deep learning helps uncover latent features from unlabeled BioMEMS sensor signals, which is critical in scenarios where manual labeling is infeasible. Autoencoders, composed of encoder–decoder architectures, extract compressed representations of continuous monitoring data [138]. Restricted Boltzmann machines, characterized by their bipartite architecture of visible and hidden layers, are adept at feature learning [139]. Deep belief networks extend this concept by stacking multiple layers and employing a pre-training phase followed by fine-tuning, enhancing their ability to model complex data [140,141]. Generative adversarial networks, on the other hand, involve a generator and discriminator in a competitive process, where the generator strives to produce data indistinguishable from real data, while the discriminator seeks to differentiate between real and generated data [142]. Together, these models provide powerful tools for exploring unstructured BioMEMS outputs and supporting tasks like clustering, dimensionality reduction, and synthetic data generation.

- Reinforcement Learning

Reinforcement learning (RL) focus on decision making through interaction with an environment. In the context of BioMEMS sensors, RL offers adaptive strategies for controlling sensing behaviors, such as dynamic sampling rates or actuator responses in closed-loop systems. However, RL can be computationally intensive for large networks, due to the extensive exploration and policy refinement required. Deep reinforcement learning (DRL), which integrates deep learning architectures, enhances the learning capability of RL models and supports complex, time-dependent sensing tasks. DRL approaches are typically categorized into value-based, policy-based, and model-based methods. Value-based methods, such as deep Q-learning, aim to approximate the value function that estimates the expected return for each state or state-action pair [143,144]. Policy-based methods directly optimize the policy to maximize the expected cumulative reward [145]. Model-based DRL focuses on learning the dynamics of the environment to predict outcomes and plan actions more effectively [146]. These methods hold promise for autonomous BioMEMS platforms, especially in wearable or implantable systems requiring real-time adaptation. However, the computational demands of DRL remain a challenge for deployment on power- and resource-limited BioMEMS platforms.

Deep learning offers a comprehensive set of tools for enhancing BioMEMS sensing systems across various data modalities. Supervised models improve classification and regression in structured tasks, while unsupervised models reveal hidden patterns in unlabeled sensor signals. Reinforcement learning introduces adaptability and autonomous decision making to closed-loop sensing environments. Collectively, these techniques support the development of intelligent BioMEMS platforms capable of real-time analysis, self-calibration, and context-aware operation.

#### 4.1.3. Big Model

- Large Language Models

Large Language Models (LLMs), including BERT [147], the GPT series [148,149,150], PaLM series [151,152], LLaMA series [153,154], PanGu series [155,156] and Deepseek series [157,158], are spearheading advancements in natural language processing, excelling in text understanding and generation across diverse tasks. These models have been extended to domains beyond text through prompt engineering and multimodal fusion. In BioMEMS systems, LLMs can be utilized to process unstructured clinical notes alongside sensor signals, generate contextual alerts, or assist in interpreting longitudinal physiological data streams by converting raw sensor outputs into human-readable insights. Furthermore, recent frameworks like DeepSeek employ self-reinforcing learning without relying on supervised labels, making them especially suitable for BioMEMS scenarios with sparse annotations.

- Large Multimodal Models

Large Multimodal Models (LMMs) extend LLMs by integrating vision, language, sound, and other modalities through unified embeddings. This architecture allows BioMEMS platforms to process hybrid inputs such as biofluid images, acoustic patterns (e.g., breath or cough sounds), or motion profiles together with textual metadata. In wearable BioMEMS applications, Transformer-based time-series models have achieved recognition accuracy of up to 99.2% for human activity monitoring [159]. This significantly outperforms traditional machine learning methods and enables real-time edge inference through tokenization and parallel processing. Backbone encoders such as Vision Transformer (ViT) [160], Whisper [161], CLIP [162], and MAE [163] provide the basis for LMMs to align physiological patterns with real-world scenarios. Their ability to perform joint representation learning makes LMMs ideal for complex biosensing tasks involving multimodal uncertainty and heterogeneous data sources.

While large-scale models such as LLMs and LMMs bring unprecedented representational capacity and multimodal reasoning capabilities, their practical deployment in BioMEMS platforms faces several challenges. First, the computational and memory requirements of such models are often incompatible with the constraints of micro-scale, battery-limited sensing systems. Second, real-time biosensing demands low-latency inference and energy-efficient computation, which remains difficult to achieve without model pruning, quantization, or edge–cloud hybrid architectures. Third, model interpretability is a pressing concern in clinical-grade applications, especially when predictions drive critical decisions.

Despite these challenges, the integration of large models with BioMEMS holds immense potential. The development of domain-adapted lightweight models and edge-tuned multimodal Transformers could enable high-accuracy sensing while preserving deployment feasibility. In addition, BioMEMS datasets can benefit from self-supervised or synthetic pretraining strategies, enhancing model generalizability in low-data scenarios. As the frontier moves from merely collecting data to extracting actionable insights, the next critical step lies in linking large model architectures with real-world sensor outputs for interpretable, predictive, and autonomous decision making.

### 4.2. AI for BioMEMS Sensors

The integration of AI with self-powered BioMEMS is driving the development of intelligent microsystems capable of real-time sensing, adaptive inference, and autonomous decision making. Advances in machine learning—from classic supervised and unsupervised algorithms to compact, on-device models—have significantly improved the accuracy, robustness, and interpretability of biosignal analysis in wearable formats [164,165]. In parallel, the convergence of energy harvesting and AI has enabled self-sustained platforms for motion recognition, physiological monitoring, and environment-aware sensing [166,167]. This section reviews key AI techniques for data interpretation, predictive modeling, and robust algorithm deployment in BioMEMS systems, highlighting recent progress and ongoing challenges in intelligent sensing at the microscale.

#### 4.2.1. Data Interpretation

As BioMEMS platforms advance toward continuous, multi-analyte sensing, the resulting data becomes increasingly complex, high-dimensional, and heterogeneous. Traditional signal processing and statistical analysis methods often struggle to extract meaningful patterns from such large and dynamic datasets. To address this, advanced AI techniques are increasingly employed to interpret real-time biosensor outputs with higher accuracy and adaptability [168]. Data interpretation typically begins with data pre-processing, which plays a critical role in improving signal fidelity and model robustness. Techniques such as denoising, baseline correction, spectral smoothing, and contrast enhancement help eliminate environmental noise and sensor artifacts. For example, accurate skin cancer detection from camera images relies on noise reduction and image restoration [169], while pre-processing methods like Savitsky–Golay smoothing improve the sensitivity of surface-enhanced Raman scattering spectra [170]. Once pre-processed, the data is ready for ML models to detect subtle, otherwise hidden patterns. In addition to enhancing raw data, synthetic data has proven valuable in augmenting BioMEMS systems, particularly in situations with missing data, such as rare diseases or underrepresented populations. Although further validation is needed for medical applications, synthetic data can reduce biases and enhance model transparency [171,172]. For example, Cycle Generative Adversarial Networks have improved non-contrast CT image segmentation, lowering the effort and cost in medical image processing [173]. Such approaches can be extended to BioMEMS data, enabling more robust model training and improved generalization across sensor platforms and patient populations.

As BioMEMS sensing systems become more integrated and autonomous, ensuring data quality, mitigating bias, and leveraging synthetic augmentation will be key to unlocking the full potential of AI-enhanced BioMEMS sensor platforms. This sets the stage for predictive analysis, where real-time insights can drive proactive diagnostics and therapeutic decisions.

#### 4.2.2. Predictive Analysis

**Figure 9:**
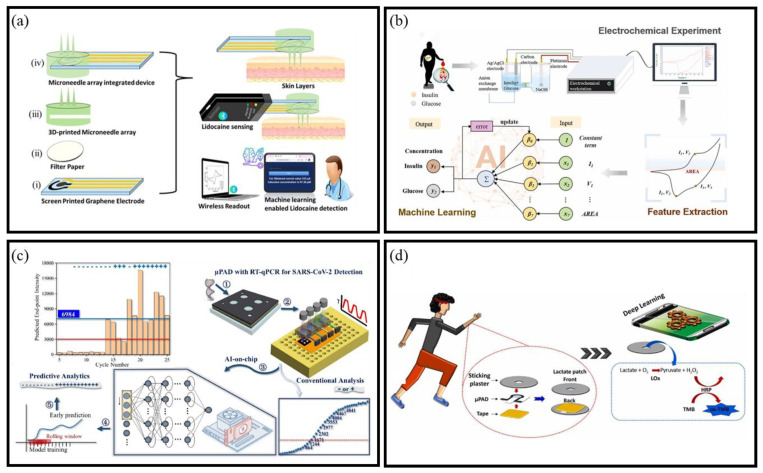
Applications of AI-enhanced BioMEMS sensors in predictive analysis. (**a**) Lidocaine detection using a wireless microneedle array with ML integration [175]. (**b**) AI-powered electrochemical detection of insulin and glucose [176]. (**c**) GRU deep learning for real-time PCR analysis with microfluidic devices [177]. (**d**) Smartphone-embedded DL for sweat lactate colorimetric analysis [178].

The integration of AI with BioMEMS sensors has significantly advanced the capabilities of predictive analysis, especially in the early diagnosis and dynamic monitoring of diseases. Traditional analytical methods often struggle to interpret high-frequency, multi-parameter data streams generated by wearable or implantable BioMEMS sensors. AI-driven predictive models enable robust detection, classification, and regression tasks with minimal human intervention, improving both accuracy and repeatability [174]. A notable example is the lidocaine detection sensor [175], which utilizes a wireless microneedle array to achieve a detection limit of 0.13 μM. This sensor integrates ML algorithms to accurately predict lidocaine concentrations (Figure 9a). Similarly, Zhao et al. introduced an AI-powered electrochemical method for detecting insulin and glucose in serum [176], which combines cyclic voltammetry with ML to offer rapid, accurate, and cost-effective multi-component analysis (Figure 9b).

In the realm of molecular diagnostics, Sun et al. [177] introduced a BioMEMS platform integrating gated recurrent unit (GRU) models with microfluidic paper-based analytical devices for PCR analysis, achieving real-time inference with minimal hardware requirements (Figure 9c). Building on this foundation, Sun et al. further advanced the field by developing a paper-based microfluidic system integrated with a Graph Neural Network [179]. This system was designed for real-time, on-device predictive analysis of nucleic acid amplification. In wearable biosensing applications, AI has been used to support real-time, multi-modal health monitoring. Veeralingam et al. designed an AI-enabled platform capable of detecting sweat pH, glucose, and skin hydration, interfacing with a microcontroller for efficient data analysis [180]. AI-powered EEG models have achieved over 90% accuracy in detecting cognitive states, such as short-term memory and emotional responses [181]. Similarly, EMG and surface EMG (sEMG) signal processing enhanced by AI improves tracking of muscle activity, supporting rehabilitation, prosthetics, and voice recognition [182]. In the sports domain, Babu et al. developed a piezoelectric nylon-11 wearable sensor integrated with ML algorithms to monitor athletic movement in real time, achieving a classification accuracy of ~97% in various training tasks [183]. Recently, Transformer-based models have been applied to BioMEMS time-series data. Karagoz et al. trained PatchTST and Crossformer on CGM signals. They achieved RMSE = 15.6 mg/dL at 30 min and up to 46.5 mg/dL at 4 h [184].

Recent studies have also demonstrated the effectiveness of advanced AI models, such as deep neural networks (DNNs), random forests, and personalized adaptive frameworks, in wearable BioMEMS-based physiological monitoring and stress detection. Barki et al. applied DNNs to biosignals acquired from the Empatica E4 wristband, achieving classification accuracies of 96% when combined with self-reports and 88% with sensor data alone [185]. Similarly, a random forest model trained on HRV and skin conductance achieved F1 scores > 65% under real-world noise and behavioral variability [186]. This confirms the feasibility of embedded AI inference in wearable BioMEMS systems. Colorimetric sensors, aided by AI, enable sensitive and rapid detection of various analytes with minimal equipment and expertise. A case in point is the work by Yuzer et al. [178], which involved a smartphone-embedded DL approach for accurate and automated colorimetric lactate analysis in sweat (Figure 9d). In the sports domain, Babu et al. developed a piezoelectric nylon-11 wearable sensor integrated with ML algorithms to monitor athletic movement in real time, achieving a classification accuracy of ~97% in various training tasks [187].

These studies collectively demonstrate how AI empowers BioMEMS platforms to move beyond simple sensing toward predictive, personalized, and autonomous diagnostics. By integrating advanced models with compact hardware, predictive analysis can be performed close to the source of data, enabling real-time decision making even in low-resource environments. Despite these promising advances, challenges remain. The generalizability of AI models across populations and sensing platforms is still limited, especially when training datasets are small or unbalanced. Furthermore, model interpretability is critical in medical contexts where regulatory transparency is required. Finally, on-device deployment of complex models demands continued innovation in low-power AI hardware and edge computing strategies. Addressing these issues will be essential for realizing fully autonomous, intelligent BioMEMS systems in next-generation diagnostics and health monitoring.

#### 4.2.3. Robustness and Interpretability in AI-Enhanced BioMEMS Sensors

The reliability and transparency of machine learning algorithms are critical for their integration into BioMEMS systems, particularly in scenarios involving continuous physiological monitoring, limited data availability, and deployment on constrained hardware platforms. Two practical concerns arise in such applications: how to ensure model robustness under diverse real-world conditions, and how to interpret model behavior to support algorithm selection and regulatory acceptance.

To mitigate issues of overfitting or underfitting, several strategies have been widely adopted. These include regularization techniques such as L2 norm constraints and dropout, as well as early stopping during training to prevent performance degradation on unseen data [188]. In small-sample settings—common in personalized sensing or rare biosignal detection—cross-validation schemes, such as k-fold or leave-one-subject-out (LOSO) validation, are necessary to assess generalizability across individuals or operating conditions [189]. Moreover, data augmentation through signal perturbation, synthetic sample generation, or domain-adaptive pretraining has proven effective in improving model stability and reducing sensitivity to signal artifacts [190].

For more complex models—such as GRUs or Transformer-based architectures—interpretability is typically achieved through post hoc techniques. Common approaches include SHAP (Shapley Additive Explanations), which quantifies the contribution of each input feature to a given prediction [191]; saliency mapping, which highlights the most influential temporal segments in biosignal sequences [183]; and attention weight visualization, which reveals the dependencies the model captures across different time steps [192]. Beyond these methods, explainable artificial intelligence (XAI) has emerged as a framework to improve transparency and trust in deep models. XAI not only enhance interpretability in materials informatics but also offer transferable strategies for increasing model explainability in biosensing applications [193].

From a practical engineering perspective, model choice often depends on the balance between performance, interpretability, and deployment constraints. Lightweight, interpretable models such as decision trees or logistic regression are often preferred in edge devices with limited computing resources or in safety-critical applications where regulatory transparency is essential. In contrast, deeper architectures may offer better accuracy and temporal pattern modeling, but require additional tools to ensure explainability and cross-platform adaptability.

In summary, ensuring algorithmic robustness through proper training protocols and maintaining interpretability through model structure or explanation techniques are essential steps toward the practical adoption of AI methods in BioMEMS platforms. These considerations guide the selection of appropriate models based on application-specific requirements for accuracy, stability, transparency, and computational cost.

### 4.3. Near-Sensor and In-Sensor Computing

With the rapid growth of the Internet of Things (IoT) and wearable health technologies, there is a rising demand for BioMEMS-based sensor systems that enable real-time signal processing, low-latency decision making, and ultra-low power consumption. However, conventional sensing architectures typically rely on backend processing, which introduces significant energy overhead, latency, and communication bottlenecks. These challenges are particularly limiting in BioMEMS applications, where device miniaturization and long-term operation are critical. To overcome these issues, near-sensor and in-sensor computing architectures have been proposed. These paradigms move data processing closer to, or even inside, the sensor unit, thereby reducing redundant data transmission and enabling more efficient localized intelligence [194].

#### 4.3.1. Neuromorphic Devices and Near/In-Sensor Computing

Neuromorphic devices such as memristors and synaptic transistors offer unique advantages for BioMEMS integration. Their low-power operation—based on microampere currents—and non-volatile memory capabilities allow them to perform basic computational tasks directly at the sensing interface [195]. These devices not only filter data but also possess the ability to perform basic processing tasks, enabling them to simulate synaptic models. This allows them to store information in resistance form and process input signals with synaptic plasticity and non-volatile characteristics, further optimizing computational efficiency at the sensor level [196].

- Near-sensor Computing

**Figure 10:**
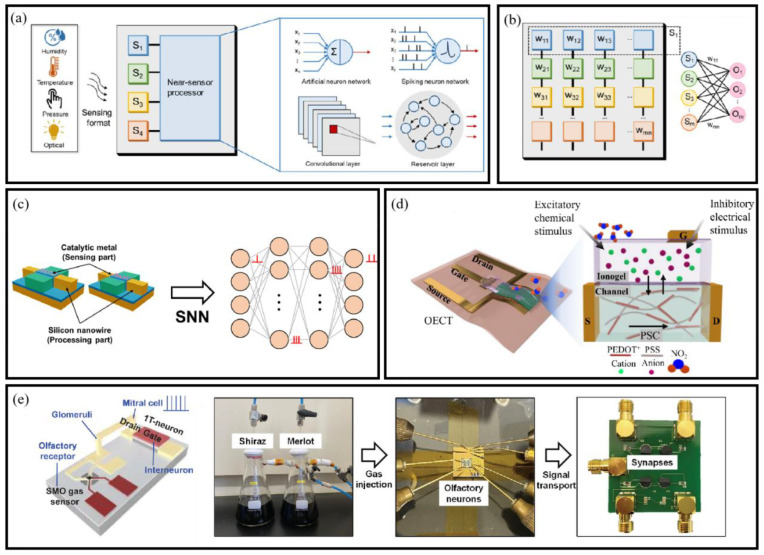
Near-sensor and in-sensor computing in BioMEMS sensors. (**a**) Near-sensor computing system design reducing computational load and energy consumption [197]. (**b**) In-sensor computing eliminating redundant data transmission [197]. (**c**) Olfactory sensory neuron using in-sensor computing for breath monitoring [198]. (**d**) Organic electrochemical transistor for NO_2_ detection [199]. (**e**) Gas sensor integrated with a neuron system for neural network-based classification [200].

In near-sensor computing, processors are placed adjacent to the sensing elements to carry out data filtering, feature extraction, and basic inference before transmitting the results. This configuration substantially reduces both data volume and power consumption, alleviating the computational burden on centralized processors (Figure 10a). For BioMEMS devices, such architecture allows initial physiological signal classification or anomaly detection to occur immediately after data acquisition, improving response time in wearable and implantable systems. Unlike traditional sensor systems that require analog-to-digital conversion, near-sensor architectures process analog signals directly, employing brain-inspired algorithms for tasks such as DNN accelerations and CNN.

- In-sensor Computing

In-sensor computing systems go further by incorporating computing functions directly within the sensor, thus eliminating the need for redundant data transmission altogether (as illustrated in Figure 10b). This approach enables the sensors not only to capture data but also to perform simple processing locally, significantly reducing the energy costs associated with sending raw data to external units. By processing analog signals directly, neuromorphic devices replace the need for ADCs, further enhancing power efficiency. Recent studies have demonstrated that integrating neuromorphic computing with near/in-sensor architectures can reduce power consumption by up to two orders of magnitude [201], showcasing the potential of these systems for applications in continuous monitoring [202].

#### 4.3.2. Near/In-Sensor Processing Applications

The implementation of near-sensor and in-sensor computing architectures has significantly advanced the functional integration of BioMEMS systems, particularly in flexible olfactory platforms. These systems, inspired by the biological olfactory mechanism, achieve molecular recognition through selective surface reactions and real-time electrical signal conversion. This design paradigm enhances molecular selectivity, sensitivity, and operational adaptability in wearable and portable sensing scenarios. Lee et al. demonstrated a silicon nanowire field-effect transistor (FET) configured as an olfactory sensory neuron, employing an in-sensor computing scheme that outperformed conventional near-sensor layouts. The FET integrated catalytic nanoparticles to detect hydrogen and ammonia in exhaled air, supporting real-time breath monitoring with spike-based output suitable for spiking neural network processing (Figure 10c).

Advancements in organic electronic materials have further enabled low-voltage gas detection. Chouhdry et al. fabricated a flexible artificial chemosensory neuronal synapse based organic electrochemical transistor, incorporating an ion-gel sensing layer. This design achieved NO_2_ detection at concentrations as low as 2.66 ppm, demonstrating high sensitivity and compatibility with flexible substrates (Figure 10d) [199]. Incorporating neuromorphic elements into the gas sensor design further improves the detection and classification capabilities of flexible olfactory systems. For instance, Han et al. further enhanced sensing resolution by embedding neuromorphic circuitry into a semiconductor metal oxide gas sensor. The system integrated a transistor-based artificial neuron, enabling analog-domain preprocessing and on-site classification of gas stimuli [200] (Figure 10e). These examples highlight the engineering potential of near/in-sensor computing in BioMEMS. They enable compact, low-latency, and energy-efficient platforms suitable for continuous environmental and physiological monitoring in real-world applications.

Compared to conventional edge AI architectures relying on CNN or Transformer-based models, in-sensor neuromorphic systems exhibit ultra-low latency and sub-mW power consumption, albeit often constrained by task-specific design and limited generalizability. For instance, spike-based classifiers embedded directly in sensor hardware can outperform MCU-based inference in terms of responsiveness and energy efficiency, especially in gas classification or olfactory applications. However, their scalability to complex physiological data streams remains a challenge. Integrating such neuromorphic approaches with adaptive learning or hybrid edge–cloud architectures may further expand their applicability across broader BioMEMS domains.

## 5. Challenges and Prospects

The integration of sustainable energy harvesting, BioMEMS sensors, and AI-driven analytics is accelerating the development of intelligent and autonomous microsystems for health monitoring, diagnostics, and environmental sensing. However, this convergence presents numerous challenges across material, computational, and system-integration domains. Addressing these multi-level constraints is crucial to enable reliable, scalable, and regulatory-compliant deployment of BioMEMS technologies in real-world conditions.

### 5.1. Energy and Computational Constraints

Achieving energy autonomy remains a major bottleneck for AI-integrated BioMEMS systems. While emerging energy harvesters such as PENGs, TENGs, MEGs, and miniaturized photovoltaic devices offer promising alternatives to conventional power sources, their energy output is inherently variable and highly sensitive to ambient fluctuations. For instance, biomechanical energy harvesting depends on human motion intensity and frequency, while solar energy varies with light conditions. This variability can lead to intermittent power supply, causing sensing interruptions and limiting the consistency of data acquisition.

In parallel, the computational and memory demands of modern AI models pose substantial integration challenges for microscale platforms. Deep learning architectures, particularly CNNs and Transformer-based models, require considerable on-chip resources for inference—often surpassing the power and memory budgets of energy-harvesting BioMEMS devices. The lack of dedicated accelerators for low-power inference exacerbates latency and throughput limitations, hindering real-time physiological analysis in wearable or implantable scenarios.

Furthermore, data scarcity is a non-negligible constraint in BioMEMS environments. Due to the difficulty of obtaining labeled, high-quality biosensor data—especially from rare or long-term pathological conditions—AI models often suffer from overfitting and poor generalization. This issue is magnified in personalized or decentralized deployments, where data heterogeneity across users or devices introduces distribution shifts that challenge model robustness.

To address these challenges, multiple strategies have been investigated. On the algorithmic front, model compression techniques, such as pruning, quantization, and knowledge distillation, can significantly reduce memory and energy requirements while retaining inference performance. In addition, spiking neural networks and neuromorphic chips have shown promise for ultra-low-power operation, mimicking biological efficiency at the circuit level.

Importantly, federated learning and transfer learning have emerged as viable solutions for privacy-preserving and data-efficient training. Federated architectures enable decentralized model updates across multiple BioMEMS nodes without centralizing raw data, which is essential for safeguarding sensitive biomedical information. Transfer learning, by leveraging knowledge from large-scale pretrained models, allows efficient adaptation to small datasets and new biosensing modalities with minimal retraining effort. These paradigms not only alleviate the data scarcity issue but also reduce communication overhead, improving scalability for real-world deployment.

Collectively, bridging the gap between power constraints, computational demand, and limited training data will require synergistic innovation across model architecture design, hardware co-optimization, and federated edge-intelligence frameworks tailored to the unique constraints of BioMEMS systems.

### 5.2. Material Challenges in Sensing and Harvesting Units

Material selection and structural design are central to the performance and reliability of BioMEMS platforms. Wearable and implantable sensors demand materials that are simultaneously lightweight, stretchable, conformal, and electrically functional. Although nanomaterials such as CNTs, MXenes, graphene, and conducting polymers exhibit excellent sensitivity and electronic tunability, they often suffer from poor mechanical robustness under cyclic deformation. Issues such as cracking, delamination, and chemical degradation are frequently observed during long-term operation in dynamic environments.

In energy harvesting units, similar challenges emerge. For instance, piezoelectric ceramics typically exhibit high energy conversion efficiency but are too brittle for flexible applications. In contrast, polymer-based harvesters offer mechanical flexibility but often deliver suboptimal power density. Recent efforts have explored hybrid structures combining multiple harvesting mechanisms—e.g., triboelectric–photovoltaic or thermoelectric–biofuel hybrids—to compensate for individual limitations. However, these systems introduce significant structural complexity and demand careful attention to interfacial compatibility, thermal stability, and miniaturized fabrication strategies.

Material aging and environmental exposure further degrade device reliability. For instance, triboelectric surfaces can experience surface wear or humidity-induced charge leakage, while MXene-based electrodes may oxidize over time. Protective coatings and material encapsulation techniques must therefore be optimized to prolong functional lifetimes.

In parallel, bio-interfacing materials—especially for implantable BioMEMS—must be rigorously evaluated for cytotoxicity, immunogenicity, and long-term tissue compatibility. While many novel materials demonstrate promising sensing or energy properties, their biocompatibility profiles remain inadequately characterized, presenting a barrier to clinical translation.

### 5.3. Integration and Deployment Barriers

Even with advanced materials and energy strategies in place, BioMEMS systems face critical integration challenges that hinder their deployment in practical biomedical or environmental settings. One of the foremost challenges is packaging. Devices must be encapsulated to prevent the ingress of moisture, ions, and biological fluids while preserving signal sensitivity and mechanical compliance. Traditional encapsulation materials such as PDMS or parylene-C provide limited long-term protection, especially under repeated strain or temperature cycling. Advanced multilayer or hybrid packaging strategies are needed to ensure hermetic sealing, chemical resistance, and sensor responsiveness.

Another essential consideration is biocompatibility. Sensing and energy harvesting components that are intended for skin contact or implantation must not trigger immune responses, inflammation, or fibrotic encapsulation. Despite recent progress, many high-performance materials used in triboelectric or piezoelectric sensors—such as fluorinated polymers or MXenes—have not undergone systematic in vivo validation. Surface modification approaches, including PEGylation or zwitterionic coatings, may help improve bioinertness but introduce additional complexity in fabrication.

From a translational standpoint, regulatory hurdles remain a significant bottleneck. Regulatory frameworks for medical devices often lag behind innovations in multifunctional nanomaterials and AI-driven platforms. Ensuring compliance with ISO and FDA standards for safety, reproducibility, and long-term performance requires rigorous testing protocols, standardized fabrication processes, and reproducible quality control—areas still underdeveloped for many emerging BioMEMS systems.

Lastly, long-term reliability in vivo poses a unique integration challenge. Physiological environments impose multifaceted stressors including mechanical vibration, biochemical degradation, temperature fluctuations, and pH changes. These stressors can lead to signal drift, corrosion, fatigue, and delamination—adversely affecting both the sensing accuracy and energy harvesting efficiency. In AI-enhanced systems, small sensor instabilities may propagate and degrade model performance over time. Therefore, future platforms must adopt self-calibration algorithms, redundancy in sensor arrays, and adaptive filtering techniques to mitigate the effects of degradation.

### 5.4. Outlook: Toward Adaptive and Intelligent BioMEMS

Progress in AI-enhanced BioMEMS will hinge on synergistic innovation across materials, energy systems, computation, and integration. On the materials front, the development of multifunctional nanocomposites—such as MXene-elastomer hybrids, ionic hydrogels, and biodegradable semiconductors—may offer new possibilities for high-sensitivity, biocompatible, and long-lasting biosensors. Combining electrical conductivity with mechanical elasticity and environmental stability remains a major design goal.

In energy harvesting, compact multimodal harvesters that integrate triboelectric, thermoelectric, and solar conversion mechanisms into a unified architecture will likely play a key role in stabilizing power supply under fluctuating ambient conditions. Such harvesters, when coupled with in-sensor computing or low-power edge AI chips, could enable real-time analytics without external power or wireless data transmission. This vision aligns with the emerging paradigm of self-powered sensing systems endowed with learning capability [203], where the integration of energy harvesting, embedded intelligence, and ethical deployment frameworks marks a key frontier for scalable, autonomous BioMEMS solutions.

From a computational standpoint, edge-oriented AI frameworks must continue to evolve. Lightweight neural architectures, on-device learning, federated training, and hardware-aware neural network design are promising directions to reduce memory and power footprints. Furthermore, robust physical-model-informed AI algorithms—capable of adapting to sensor drift and user-specific variations—could enhance system interpretability, resilience, and personalization.

Finally, integration with digital twin technologies may allow real-time simulation and visualization of physiological and environmental dynamics based on BioMEMS data streams. This convergence of physical sensing, AI analytics, and virtual modeling will open new frontiers in personalized medicine, remote diagnostics, and intelligent environmental monitoring.

## 6. Conclusions

This review highlights the emerging integration of BioMEMS sensors with sustainable energy harvesting and artificial intelligence, forming a promising framework for autonomous, continuous, and intelligent sensing. MEGs, PENGs, and TENGs offer diverse strategies for self-powered operation, each with distinct mechanisms and material requirements. Their combination with flexible architectures enables effective energy harvesting in wearable and implantable formats.

Simultaneously, AI models—including traditional machine learning, deep learning, and large-scale neural networks—have significantly improved data interpretation, signal classification, and predictive analysis. When applied to BioMEMS platforms, these models enable the robust detection of physiological and biochemical targets, even under noisy or resource-constrained conditions. Moreover, the development of near-sensor and in-sensor computing technologies, particularly those based on neuromorphic elements, further addresses the challenge of real-time, low-power processing.

Despite these advances, several limitations remain, including unstable power output, limited material durability, and the high computational cost of on-device inference. Future progress requires the co-optimization of energy harvesters, sensor materials, and embedded AI frameworks. Strategies such as multimodal energy systems, domain-adaptive machine learning, and the integration of edge intelligence will be key to realizing practical, scalable BioMEMS solutions.

By bridging sustainable power supply, smart analytics, and microscale sensing technologies, the next generation of BioMEMS systems is expected to play a transformative role in personalized healthcare, human–machine interaction, and environmental intelligence.

## Figures and Tables

**Table 1:** Comparison of nanomaterials for BioMEMS sensors.

Material Type	Sensing Performance	Stability	Biocompatibility	Electrical Conductivity	Processability	Representative Applications
0D Metal Nanoparticles	High sensitivity due to large surface area; excellent for molecular recognition	Prone to aggregation; requires stabilizers	Good (especially AuNPs)	Moderate to high	Easy surface functionalization; scalable	Antibody/antigen biosensors, dopamine sensing [13,14]
0D Quantum Dots	Tunable photoluminescence; excellent for optical sensors	Sensitive to oxidation and photobleaching	Generally good (especially carbon-based)	Low to moderate	Requires encapsulation or passivation	Cholesterol and glioma sensing [17,18]
1D Nanotubes	Excellent electrochemical response; fast electron transfer	Chemically stable, but long-term biocompatibility varies	Moderate to good (depends on functionalization)	High	Complex alignment and purification	Glucose sensors, drug detection [20,21]
1D Nanowires	High aspect ratio; excellent for label-free sensing	Good structural stability	High (especially silicon-based)	Moderate	Compatible with top-down and bottom-up fabrication	C-reactive protein and DNA detection [25,27]
2D Graphene	High carrier mobility; ultrathin interface enhances sensitivity	Chemically stable, but functionalization affects performance	Good (especially GO)	High	Excellent lithographic compatibility	H_2_O_2_, cholesterol, sweat glucose sensors [30,31,32]
2D MXenes	Strong signal amplification, fast response	Sensitive to oxidation in ambient air	Excellent; hydrophilic surface supports immobilization	Very high	Requires etching and passivation	Paraoxon, phenol, H_2_O_2_ detection [34,35,36]

**Table 2:** Essential performance metrics comparison of PENGs, TENGs, and MEGs.

Working Mechanism	Materials	Open-Circuit Voltage	Output Power	Current Density	Power Density	Refs.
PENG	Mxene/black phosphorus			6.94 mA cm^−2^	2.22 mW cm^−2^	[110]
PENG	PVDF/ZnO/rGO				138 ± 2.82 μW/cm^3^	[111]
PENG	PVDF/BT	4 V			87 μW cm^−3^	[56]
PENG	PVDF/ZnO	84.5 V	0.46 mW		41.02 μW/cm^2^	[112]
PENG	PVDF-TrFE/MXene	1.5 N (at 20 N)			3.64 mW/m^2^ (at 20 N)	[61]
PENG	PMN-PT	20 V (series); 12 V (parallel)				[113]
PENG	PMN-PT	8.1 V	6.9 μW			[114]
TENG	PTFE/Cu		40 μW			[115]
TENG	PDMS/Cu				1768.2 mW m^−2^ (at 1200 N)	[116]
TENG	PVDF-HFP/AgNWs/Mn-BNT-BT	2170 V			47 W/m^2^	[117]
TENG	PTFE/Al	65.2 V			110 mW m^−2^ (at 100 MΩ)	[118]
TENG	POM/PTFE	6.0 V			2200 mW/m^3^ (at 100 MΩ)	[119]
MEG	Mxene/PAM	600 mV		1160 μA cm^−2^	24.8 μW cm^−2^	[120]
MEG	BPF	0.95 V (at 25% RH and 25 °C)			5.52 μW cm^−2^ (at 85% RH)	[98]
MEG	ANF/MXene/CNT	0.42 V			1.577 µW cm^−2^ (at 100 KΩ.)	[121]
MEG	Nonwoven fabrics/HCNTs/PVA	0.56 V (at 42% RH and 21.5 °C)			105.7 μW cm^−3^	[109]
MEG	Nanowires/Mg/Al	37 mV (Mg; at 25% RH); 51 mV (Al; at 25% RH)			20.8 mW cm^−3^ (Mg), 39.3 mW cm^−3^ (Al)	[104]
MEG	SMEG/Q-CNF/CMC/SWCNT	668 mV		6.4 μA	0.871 μW cm^−2^ (at 90% RH)	[122]

## Data Availability

No new data were created or analyzed in this study.

## Abbreviations

The following abbreviations are used in this manuscript:

MEGs	Moisture Electricity Generators
PENGs	Piezoelectric Nanogenerators
TENGs	Triboelectric Nanogenerators
BioMEMS	Bio-microelectromechanical systems
LLMs	Large Language Models
DNNs	Deep Neural Networks
NLP	Natural Language Processing
KNNs	K-Nearest Neighbors
LDA	Linear Discriminant Analysis
GO	Graphene Oxide
MXenes	Two-dimensional transition metal carbides, nitrides, and carbonitrides
CB	Carbon Black
MNPs	Metal Nanoparticles
QDs	Quantum Dots
CNTs	Carbon Nanotubes
PDMS	Polydimethylsiloxane
LOx	Lactate Oxidase
UA	Uric Acid
LLaMA	Large Language Model for Automatic Meta-Analysis
ViT	Vision Transformer
CLIP	Contrastive Language-Image Pre-training
MAE	Masked Autoencoder
GRU	Gated Recurrent Unit
